# MiR-21 in Lung Transplant Recipients With Chronic Lung Allograft Dysfunction

**DOI:** 10.3389/ti.2021.10184

**Published:** 2022-01-13

**Authors:** Naofumi Miyahara, Alberto Benazzo, Felicitas Oberndorfer, Akinori Iwasaki, Viktoria Laszlo, Balasz Döme, Mir Ali Hoda, Peter Jaksch, Walter Klepetko, Konrad Hoetzenecker

**Affiliations:** ^1^ Department of Thoracic Surgery, Medical University of Vienna, Vienna, Austria; ^2^ Department of General Thoracic, Breast, and Pediatric Surgery, Fukuoka University Hospital, Fukuoka, Japan; ^3^ Department of Pathology, Medical University of Vienna, Vienna, Austria

**Keywords:** mir-21, chronic lung allograft dysfunction, bronchiolitis obliterans syndrome, restrictive allograft syndrome, lung transplantation

## Abstract

**Background:** Micro-RNA-21 (miR-21) is a post-translational regulator involved in epithelial-to-mesenchymal transition (EMT). Since EMT is thought to contribute to chronic lung allograft dysfunction (CLAD), we aimed to characterize miR-21 expression and distinct EMT markers in CLAD.

**Methods:** Expression of miR-21, vimentin, Notch intracellular domain (NICD) and SMAD 2/3 was investigated in explanted CLAD lungs of patients who underwent retransplantation. Circulating miR-21 was determined in collected serum samples of CLAD and matched stable recipients.

**Results:** The frequency of miR-21 expression was higher in restrictive allograft syndrome (RAS) than in bronchiolitis obliterans syndrome (BOS) specimens (86 vs 30%, *p* = 0.01); Vimentin, NICD and p-SMAD 2/3 were positive in 17 (100%), 12 (71%), and 7 (42%) BOS patients and in 7 (100%), 4 (57%) and 4 (57%) RAS cases, respectively. All four markers were negative in control tissue from donor lungs. RAS patients showed a significant increase in serum concentration of miR-21 over time as compared to stable recipients (*p* = 0.040).

**Conclusion:** To the best of our knowledge this is the first study highlighting the role miR-21 in CLAD. Further studies are necessary to investigate the involvement of miR-21 in the pathogenesis of CLAD and its potential as a therapeutic target.

## Graphical Abstract

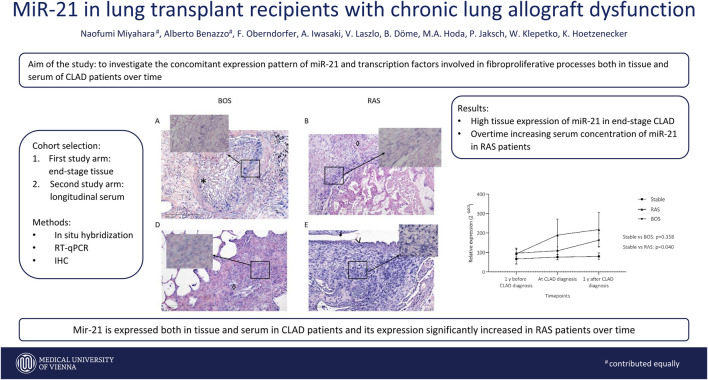

## Introduction

Chronic lung allograft dysfunction (CLAD) represents the main cause of long-term morbidity and mortality after lung transplantation (LTx). CLAD can manifest either as bronchiolitis obliterans syndrome (BOS) or restrictive allograft syndrome (RAS), mixed phenotype or as an undefined entity. CLAD affects up to 50% of lung transplant recipients within 5 years (1). Although significant efforts have been made to unravel the pathophysiology of CLAD, the main causative factors as well as therapeutic targets are still elusive. After an initial epithelial and endothelial injury, a series of immune and inflammatory stimuli trigger the activation of different pro-fibrogenic processes (2). These include activation of specific signaling pathways, activation of resident mesenchymal stromal cells and macrophages, proliferation of myofibroblasts, deposition of collagen by fibroblasts as well as epithelial-to-mesenchymal transition (EMT) (3, 4). Activation of transforming growth factor-β1 (TGF-β1), tyrosine kinase, Notch, and integrin signaling pathways leads to the deposition of extracellular matrix and to the phenotypic transition of epithelial cells into mesenchymal cells. MicroRNA-21 (miR-21) is a post-translational regulator of several signaling pathways involved in EMT. Moreover, it is a central regulator of the TGF-β1/SMAD (5) and is highly expressed during the development of lung fibrosis (6).

This study aimed to investigate the concomitant expression pattern of miR-21 and transcription factors involved in fibroproliferative processes both in tissue and serum of CLAD patients over time. The rationale of this study was to explore the role of miR-21 as predictive biomarker and potential therapeutic target against fibroproliferative derangements observed in CLAD development.

## Materials and Methods

### Cohort Selection and Tissue Samples

This work was based on two study arms. The first study arm included tissue specimens from explanted CLAD allografts at time of retransplantations. The second study arm used prospectively collected serum samples from lung transplant recipients in follow-up at our institution. This study was approved by the Institutional Review Board of the Medical University of Vienna, Austria (EK-No.2106/2017).

#### First Study Arm

All patients who received retransplantation at the Medical University of Vienna, for CLAD between 2009 and 2017 were included in this single-center study (17 BOS and seven RAS patients). Mixed and undefined phenotypes were excluded. Moreover, patients who changed their CLAD phenotype between onset and retransplantation were not included in the study. Formalin-Fixed Paraffin-Embedded (FFPE) tissue specimens from explanted lung allografts were used for quantitative real-time PCR and histopathological analysis. Specimens for IHC and ISH were obtained from the most affected of the five explanted lobes. Resected donor lung parenchyma obtained from size-reduced transplantations was used as control tissue (*n* = 4, female *n* = 3, median age = 42 ± 10). Control tissue was histologically analyzed and, if free from any parenchymal diseases, was used as healthy control.

#### Second Study Arm

A case-control cohort was identified nested within a longitudinal cohort of lung transplant recipients at the Medical University of Vienna, who consented for storage of serum. At our institution a total of 710 patients consented for prospective storage of biological samples including serum, plasma and BAL for scientific purposes from 2009 to 2014. Among them, 213 recipients developed CLAD during follow-up. Controls were chosen among 497 CLAD-free recipients by matching according to gender, age, underlying diagnosis, type of transplantation and type of induction therapy. Thirty patients in each group were initially identified, however only for 25 CLAD and 26 non-CLAD recipients, serum samples were available for all three time points defined by the study protocol. MiR-21 concentration was measured in serum samples of 51 lung recipients (13 BOS, 12 RAS, 26 stable patients) at the three defined timepoints. The timepoints for CLAD group were: 1 year before CLAD diagnosis or matched, at the time of CLAD diagnosis and 1 year after CLAD diagnosis. The first time point of serum sampling in the control arm was matched to the time point in the CLAD arm. This approach was chosen to address possible differences in miR21 expression related to the time passed since LTx. Antibody-mediated rejection (AMR) was defined according to the last ISHLT recommendations (7). Higher grade acute cellular rejection (ACR) and lymphocytic bronchiolitis (LB) were defined as ≥ A2 and B2, respectively (8). Cumulative A and B scores are the sum of all A and B scores divided by the number of biopsies performed in the follow-up per patient. BOS and RAS were diagnosed according to the most recent ISHLT classification (9) determined by two transplant physicians. At time of sampling, no patient had signs or diagnosis of ACR, AMR, infection systemic inflammation. Patients with an established CLAD diagnosis received azithromycin (250 mg three times a week) until retransplantation. Seven patients underwent extracorporeal photopheresis (ECP), due to further deterioration. In all patients, ECP was not started before 1 year after CLAD diagnosis (the third timepoint for serum collection).

### Quantitative Real-Time PCR (RT-qPCR) of miR-21

For quantification of tissue miR-21, RNA was extracted by 5 × 10 µm tissue sections from FFPE blocks using miRCURY™ RNA Isolation Kit—FFPE samples (Exiqon, Vedbaek, Denmark), according to the manufacturer’s instructions. cDNA was synthesized using High-Capacity cDNA Reverse Transcription Kit (ThermoFisher Scientific, Waltham, Massachusetts, United States) and individual miRNA-specific RT primers (Exiqon, Vedbaek, Denmark). Micro-RNA levels were quantified in duplicates from 4 μl cDNA, with SYBR Green PCR Master Mix and specific primers of the miRCURY LNA™ miRNA PCR assay, using the following settings on an Applied Biosystems™ 7500 Fast Real-Time PCR system (Thermo Fisher Scientific, United States): 2 min, 50°C; 10 s, 95°C; 40 cycles of 10 s, 95°C; 1 min, 60°C.

Serum RNA, including miRNAs, was extracted from 200 μl patient serum, by using the miRNeasy Serum/Plasma Advanced kit (QIAGEN, Germany) according to the manufacturer’s instructions. cDNA was synthesized from 2.5 μl of serum-RNA by using individual miRNA-specific RT primers contained in the 5× miRCURY RT reaction buffer and 10× miRCURY RT enzyme mix (QIAGEN, Germany), by using the following thermal cycler conditions: 60 min, 42°C; 5 min, 95°C. Circulating miRNA levels were quantified in duplicate from 4 μl cDNA, with SYBR Green PCR Master Mix and specific primers of the miRCURY LNA™ miRNA PCR assay, using the amplification condition explained above.

RT-qPCR data were analyzed via the comparative threshold cycle (Ct) method [6]. The concentration of circulating miR-21 was expressed as 2^−∆∆Ct^ and compared with to control samples.

### *In Situ* Hybridization (ISH)

ISH was performed according to the manufacturer’s protocol (QIAGEN, Hilden, Germany) (10), with some modifications. A double-DIG labeled miRCURY LNA™ microRNA detection probe with the sequences 5′-TCA​ACA​TCA​GTC​TGA​TAA​GCT​A-3′ and a U6 probe (positive control) was used, while a scrambled probe served as negative control. U6 small nuclear RNA (snRNA) is a noncoding RNA transcript used in pre-mRNA splicing expressed in all cells. Therefore, ISH for U6 revealed an intense signal in cell nuclei. Tissue sections (6 μm thick) were deparaffinized in descending ethanol solutions (99, 96, 70%) and digested with Proteinase-K (15 μg/ml) for 30 min at 37° using an Abbott hybridizer System. Then, LNATM probes were denatured and diluted in QIAGEN ISH buffer. Hybridization with 40 nM MiR21 probe, 1 nM U6 probe and 40 nM scramble probe was performed at 50°C for 60 min followed by stringent washes in 5 × SSC, 1 × SSC and 0.2 × SSC at 50°C and blocking. The digoxigenins were recognized by a specific anti-DIG antibody conjugated with Alkaline phosphatase (AP). Samples were stained with freshly prepared NBT/BCIP substrate reagent containing 0.2 mM Levamisole (2 h at 30°C) and slides incubated with KTBT buffer. Nuclear Fast Red was used as counter stain. Sections were analyzed microscopically.

MiR-21 intensity in fibroblast cytoplasm and extracellular matrix was retained for scoring purposes with a minimum cut-off at 10% of cells. Cases were classified as: 0 = negative or faint expression; 1 +: low expression (<25%); 2 +, moderate expression (25–50%); 3 +, strong expression (>50%). Cases with a score of three were regarded as positive in Kaplan-Meier curves.

### Immunohistochemistry (IHC)

All stainings were performed on sections of 2–3 µm thickness. IHC was conducted according to a standard protocol using the following antibodies: Vimentin (Clone V9, Biocare medical) at dilutions of 1:300, Notch intracellular domain (NICD) (Clone A-8, Santa Cruz Biotechnology) at dilutions of 1:50, p-Sma and Mad-related protein (SMAD) 2/3 (Clone C-8, Santa Cruz Biotechnology) at dilutions of 1:100, β-catenin (Clone 14, BD Transduction laboratories) at dilutions of 1:100 and E-cadherin (Clone NCH-38, DAKO) at dilutions of 1:2. Staining was either performed with a BenchMark Ultra or a BenchMark XT (Ventana, Tucson, AZ). Negative and positive controls demonstrated appropriate immunolabeling for each staining.

The proportion of epithelial cells or fibroblasts and extracellular matrix that were positive for each marker was classified as: 0 = negative or faint expression; 1 +: low expression (<25%); 2 +, moderate expression (25–50%); 3 +, strong expression (>50%). IHC and ISH were reviewed and scored by two independent researchers (A.B., F.O.), and cases with at least 1 + were regarded as positive.

### Statistical Analyses

Categorical variables are reported as percentage, continuous variables as mean (min-max). A X^2^ test, Fisher exact test or one-way ANOVA test was used to test differences. Correlations were quantified with Pearson’s correlation coefficient. The values of serum miR-21 (2^−∆∆Ct^) for CLAD patients and stable patients were compared by 2-way repeated measure ANOVA test to evaluate significant trends over time. A *p*-value of 0.05 or less was considered statistically significant. All the statistical analyses were conducted with IBM SPSS Statistics version 25 (IBM, Chicago, IL) and graphics were designed with GraphPad Prism 8.

## Results

### Patient Demographics

Clinical characteristics of the 24 patients included in the histological analysis are shown in Table 1. Seventeen (70%) patients were female, age at transplantation was 26 ± 11 years and at CLAD diagnosis 33 ± 12 years. The most frequent underlying diagnosis was cystic fibrosis (9, 38%), followed by interstitial lung disease (7, 29%) and idiopathic pulmonary arterial hypertension (4, 16%). Thirteen (54%) patients did not receive any induction therapy, 7 (29%) patients received rabbit anti-thymocyte globulins and 4 (17%) alemtuzumab. Fifteen (63%) patients were treated by azithromycin at time of CLAD onset, and twelve (50%) underwent extracorporeal photopheresis.

**TABLE 1:** Patient demographics.

		Study arm—tissue			Study arm—Serum			
Characteristics		BOS^a^ *n* = 17	RAS^b^ *n* = 7	*p*-value	BOS (*n* = 13)	RAS (*n* = 12)	Controls (*n* = 26)	*p*-value
Female		12 (70)	5 (71)	0.967	7 (53%)	3 (25%)	14 (53%)	0.21
Age, year (mean ± SD^c^)		27.3 ± 11.7	26.3 ± 11.8	0.907	46 ± 14	52 ± 10	46 ± 13	0.41
Underlying diagnosis	COPD^d^	1 (5.9%)	1 (14.3%)	0.207	5 (39%)	11 (92%)	13 (50%)	0.332
	Fibrosis^e^	7 (41.2%)	0		3 (23%)	0	2 (8%)	
	iPAH^f^	3 (17.6%)	1 (14.3%)		2 (15%)	0	1 (4%)	
	CF^g^	5 (29.4%)	4 (57.1%)		3 (23%)	1 (8%)	5 (19%)	
	Others	1 (5.9%)	1 (14.3%9		0	0	5 (19%)	
CMV^h^ risk	D+/R−	3 (17.5%)	3 (42.9%)	0.409	7 (54%)	5 (42%)	4 (15%)	0.087
	D+/R+	9 (52.9%)	2 (28.5%)		2 (15%)	3 (25%)	9 (46%)	
	D−/R+	2 (14.8%)	2 (28.5%)		3 (23%)	3 (25%)	8 (30%)	
	D−/R−	2 (14.8%)	0		1 (8%)	1 (8%)	5 (19%)	
Primary transplant type	Single	4 (23%)	0 (0%)	0.159	2 (15%)	1 (8%)	2 (8%)	0.753
	Double	13 (76%)	7 (100%)		11 (85%)	11 (92%)	24 (92%)	
Intraoperative VA ECMO^i^		11 (64.7%)	3 (42.9%)	0.324	5 (39%)	3 (25%)	13 (50%)	0.530
Prolonged postoperative VA ECMO		6 (35.3%)	1 (14.3%)	0.303	1 (8%)	1 (8%)	4 (15%)	0.712
Induction therapy	No induction	12 (70.6%)	1 (14.3%)	0.041	10 (77%)	10 (84%)	11 (42%)	0.333
	rATG^j^	3 (17.6%)	4 (57.1%)		3 (23%)	1 (8%)	11 (42%)	
	Alemtuzumab	2 (11.8%)	2 (28.6%)		0	1 (8%)	4 (16%)	
Higher grade ACR^k^ (A ≥ 2)		4 (23.5%)	1 (14.1%)	0.612	2 (15%)	1 (8%)	6 (23%)	0.588
Time to higher grade ACR from time of LTx^l^, months (mean ± SD)		12 ± 14	0.1	0.500	26 ± 18	14	6.5 ± 4	0.064
Higher grade LB^m^ (B ≥ 2)		7 (41.2%)	3 (42.3%)	0.939	6 (46%)	2 (17%)	4 (15%)	0.114
Time to higher grade LB from time of LTx^l^, months (mean ± SD)		12 ± 15	0.7 ± 1.2	0.250	21 ± 17	9 ± 12	23 ± 28	0.370
Clinical AMR^n^		2 (11.8%)	1 (14.3%)	0.865	0	0	0	—
Time to higher grade AMR from time of LTx^l^, months (mean ± SD)		94 ± 63	18	0.667	—	—	—	—
Cumulative A score		0.23 ± 0.20	0.14 ± 0.25	0.114	0.33 ± 0.24	0.24 ± 0.025	0.19 ± 0.22	0.489
Cumulative B score		0.60 ± 0.42	0.54 ± 0.62	0.534	0.85 ± 0.45	0.62 ± 0.25	0.51 ± 0.28	0.094
Azythromycin therapy for CLAD^o^		10 (58.8%)	5 (71.4%)	0.562	9 (69%)	12 (100%)	0	0.036
Extracorporeal photopheresis for CLAD		8 (50%)	4 (57.1%)	0.752	6 (46%)	1 (8%)	0	0.035
Time to CLAD from time of LTx^l^, months (mean ± SD)		34.4 ± 34.3	52.3 ± 54.1	0.494	59.1 ± 37.5	38.4 ± 29.3	—	0.21
Time to Re-LTx from time of LTx^l^, months (mean ± SD)		70.4 ± 58.8	68 ± 51.7	0.852	—	—	—	—

a Bronchiolitis obliterans syndrome.

b Restrictive allograft syndrome.

c Standard deviation.

d Chronic obstructive pulmonary disease.

e Idiopathic pulmonary fibrosis.

f idiopathic pulmonary arterial hypertension.

g Cystic fibrosis.

h Cytomegalovirus.

i Veno-Arterial extracorporeal membrane oxygenation.

j Rabbit anti-thymocyte globulin.

k Acute cellular rejection.

l Transplantation.

m Lymphocytic bronchiolitis.

n Antibody-mediated rejection.

o Chronic lung allograft dysfunction.

Clinical characteristics of the 51 patients included in the serum analysis are detailed in Table 1. Gender and age were equally distributed between the CLAD and control group with 10 (40%) vs 14 (53%) females and 49 ± 14 vs 46 ± 13 years. In both groups, the most frequent underlying diagnosis was COPD (16, 64% vs 13, 50%), followed by cystic fibrosis (4, 16% vs 5, 19%). All twelve patients with RAS received azithromycin compared to nine (69%) patients with BOS (*p* = 0.036). Extracorporeal photopheresis was more often used in BOS (6, 46%) than in RAS (1, 8%) patients (*p* = 0.035).

### MiR-21 Expression in CLAD and Control Tissue

**FIGURE 1:**
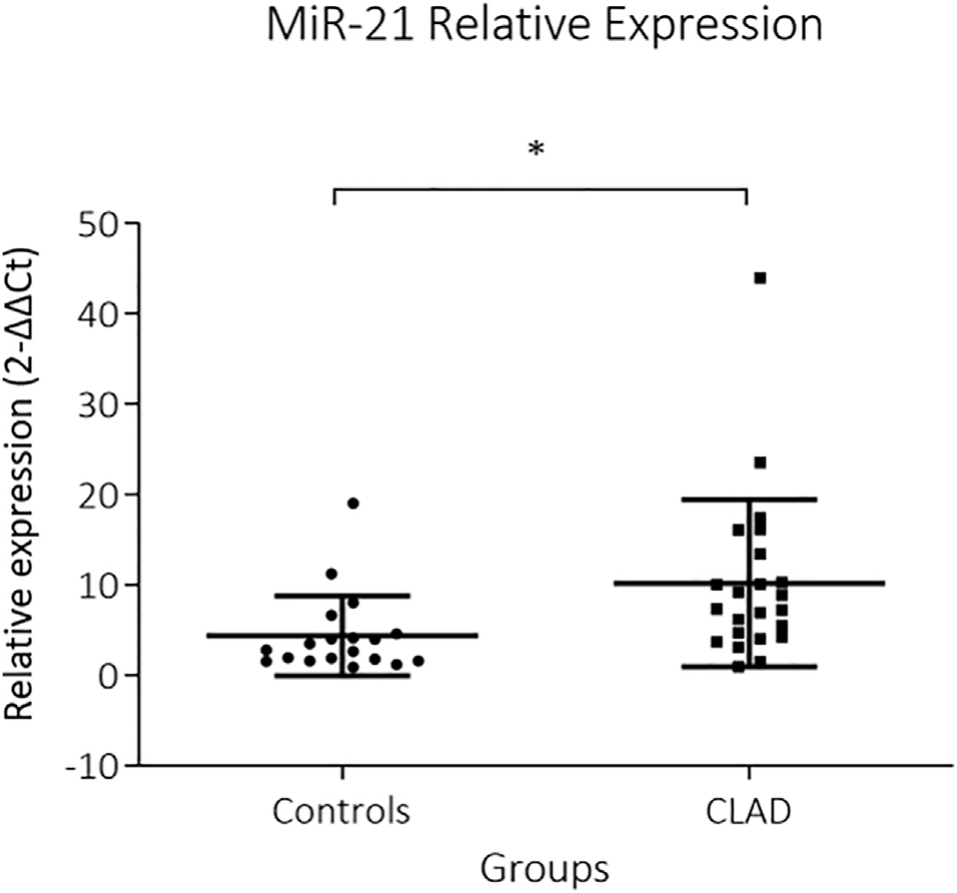
Relative expression of miR-21 in CLAD allografts. Expression of miR-21 in explanted lung allografts and normal lung tissue is shown. MiR-21 is strongly expressed in CLAD tissue whereas it is low in control tissue (*p* = 0.002). MiR-21 expression is presented as 2^−∆∆Ct^ values (Mean + Standard error).

**FIGURE 2:**
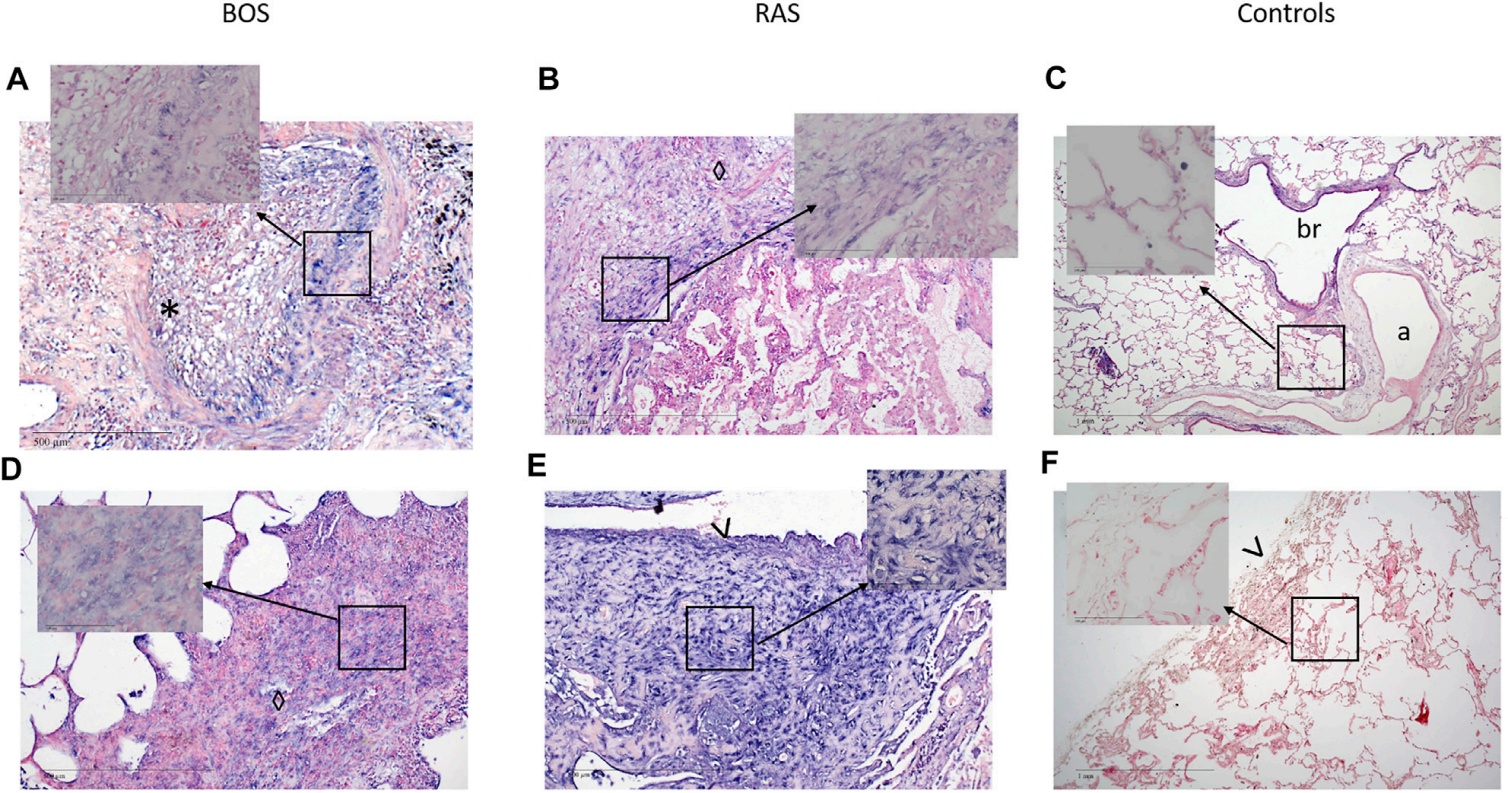
Representative images of miR-21 *in situ* hybridization in BOS and RAS lesions and in healthy lung tissue. Images of a completely obliterated bronchiole **(A)** and interstitial **(D)** staining of miR-21 (blue) in a BOS sample (original magnification **(A,D)** = ×10, insert original magnification **(A,D)** = ×40). Interstitial **(B)** and subpleural **(E)** fibrosis with architectural distortion in a RAS sample, showing diffuse positive miR-21 staining (original magnification **(B,E)** = ×10, insert original magnification **(B,E)** = ×40). Images of healthy lung parenchyma **(C)** and pleura **(F)** of control lungs (original magnification **(C,F)** = ×4, insert original magnification **(C,F)** = ×40). miR-21 = microRNA-21, ISH = *in situ* hybridization, *, obliterated bronchiole; ◊, interstitial fibrosis; >, pleura; a, artery; br, bronchiole. Nuclear Fast Red was used as counter stain.

MiR-21 was significantly upregulated in CLAD tissue (2^−∆∆Ct^: 10.1 ± 9.2 vs 4.3 ± 4.4, *p* = 0.002) (Figure 1). RT-PCR data were supplemented by *in situ* hybridization of miR-21, in order to identify distinct expression patterns within the lung parenchyma. Five (30%) BOS samples and 6 (86%) RAS samples showed positive ISH staining for miR-21 (Figure 2; Table 2). In BOS samples, positivity of miR-21 was mainly prevalent in peribronchiolar fibroblasts, in the bronchiolar epithelium as well as in the myofibroblasts of bronchiolar obliterative lesions. In RAS lesions, miR-21 staining was most commonly found in parenchymal fibroblasts and the extracellular matrix (ECM) as well as in the interlobular septa. In both BOS and RAS samples, macrophages showed a positive miR-21 staining (Supplementary Figure S1). Of note, miR-21 was mostly absent in control lung specimens, however slight positivity of miR-21 was observed in bronchiolar epithelium.

**TABLE 2:** Expression of EMT markers and miR-21 in BOS and RAS.

		Grading							
		Intra/Peribronchial				Interstitium			
		0	1+	2+	3+	0	1+	2+	3+
BOS^a^	Vimentin	0 (0)	4 (23.5)	9 (53)	4 (23.5)	8 (47)	4 (23)	3 (18)	1 (6)
	NICD^b^	5 (29)	9 (53)	0 (0)	3 (18)	8 (47)	5 (29)	3 (18)	0 (0)
	SMAD^c^	10 (59)	7 (41)	0 (0)	0 (0)	10 (59)	5 (29)	1 (6)	0 (0)
	β-catenin	17 (100)	0 (0)	0 (0)	0 (0)	17 (100)	0 (0)	0 (0)	0 (0)
	E-cadherin	17 (100)	0 (0)	0 (0)	0 (0)	17 (100)	0 (0)	0 (0)	0 (0)
	miR-21	12 (70)	3 (18)	2 (12)	0 (0)	9 (53)	4 (23)	1 (6)	2 (12)
RAS^d^	Vimentin	1 (14)	5 (72)	1 (14)	0	0 (0)	2 (28)	3 (42)	2 (28)
	NICD	2 (29)	4 (57)	1 (14)	0 (0)	3 (42)	2 (28)	0	2 (28)
	SMAD	2 (29)	4 (57)	1 (14)	0 (0)	3 (42)	3 (42)	0 (0)	1 (14)
	β-catenin	7 (100)	0 (0)	0 (0)	0 (0)	7 (100)	0 (0)	0 (0)	0 (0)
	E-cadherin	7 (100)	0 (0)	0 (0)	0 (0)	7 (100)	0 (0)	0 (0)	0 (0)
	miR-21	0 (0)	2 (28.5)	2 (28.5)	3 (43)	1 (14)	0 (0)	2 (28)	4 (57)

a Bronchiolitis obliterans syndrome.

b Notch intracellular domain.

c Sma and Mad-related protein.

d Restrictive allograft syndrome.

### Expression of EMT Markers

**FIGURE 3:**
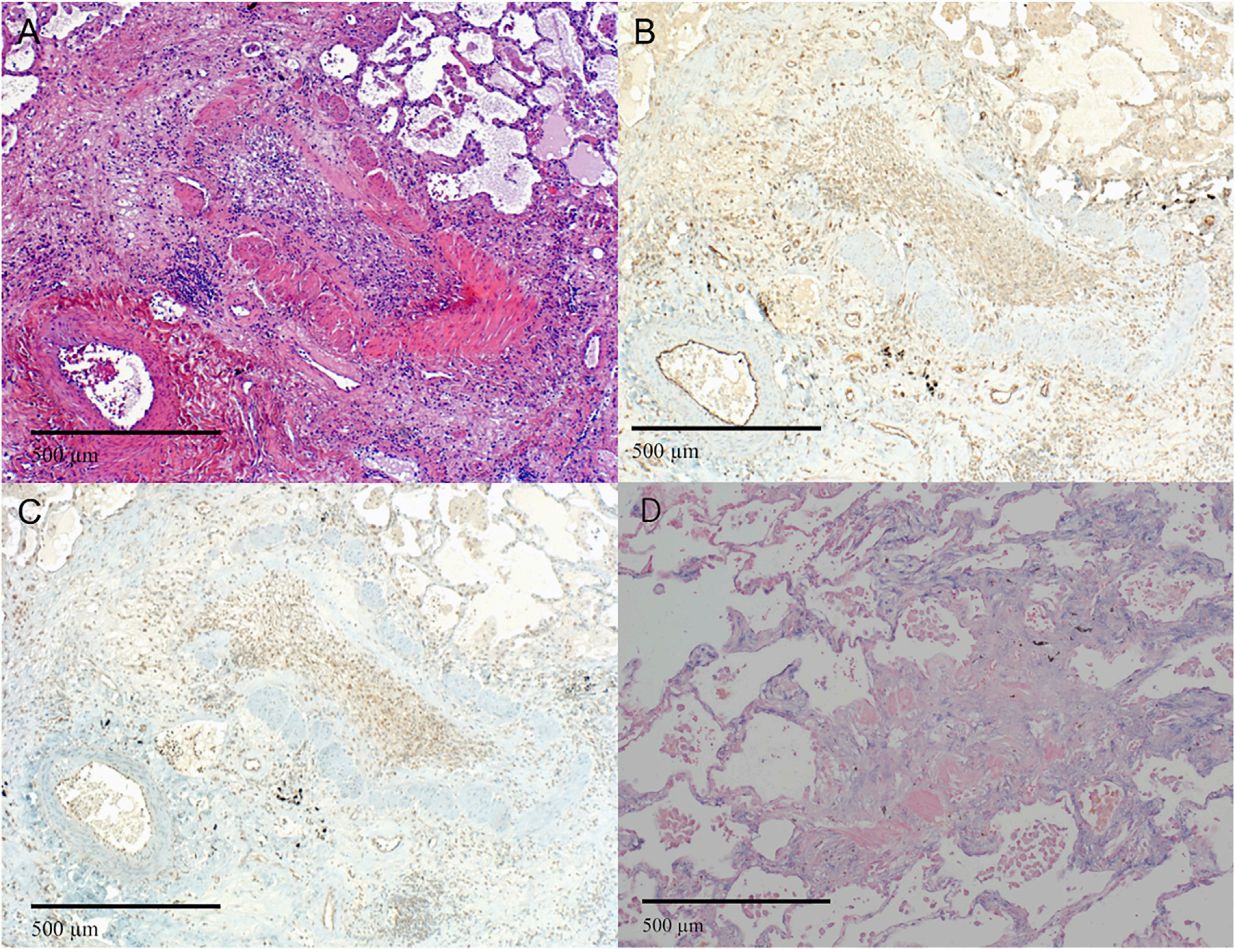
Representative images of bronchiolitis obliterans in BOS. **(A–D)** Completely obliterated bronchioles. **(A)** H&E staining of a bronchiole showing luminal loose fibrous connective tissue with scattered chronic inflammatory cells and fibroblasts. **(B and C)** Immunohistochemistry of the same area as in **(A)** showing **(B)** NOTCH1 and **(C)** pSMAD2,3 expression of the lesion. **(D)** miR-21 ISH shows expression of miR-21 in fibroblasts. (A-D original magnification ×10). H&E = Hematoxylin and Eosin; miR-21 = microRNA-21, ISH = *in situ* hybridization.

**FIGURE 4:**
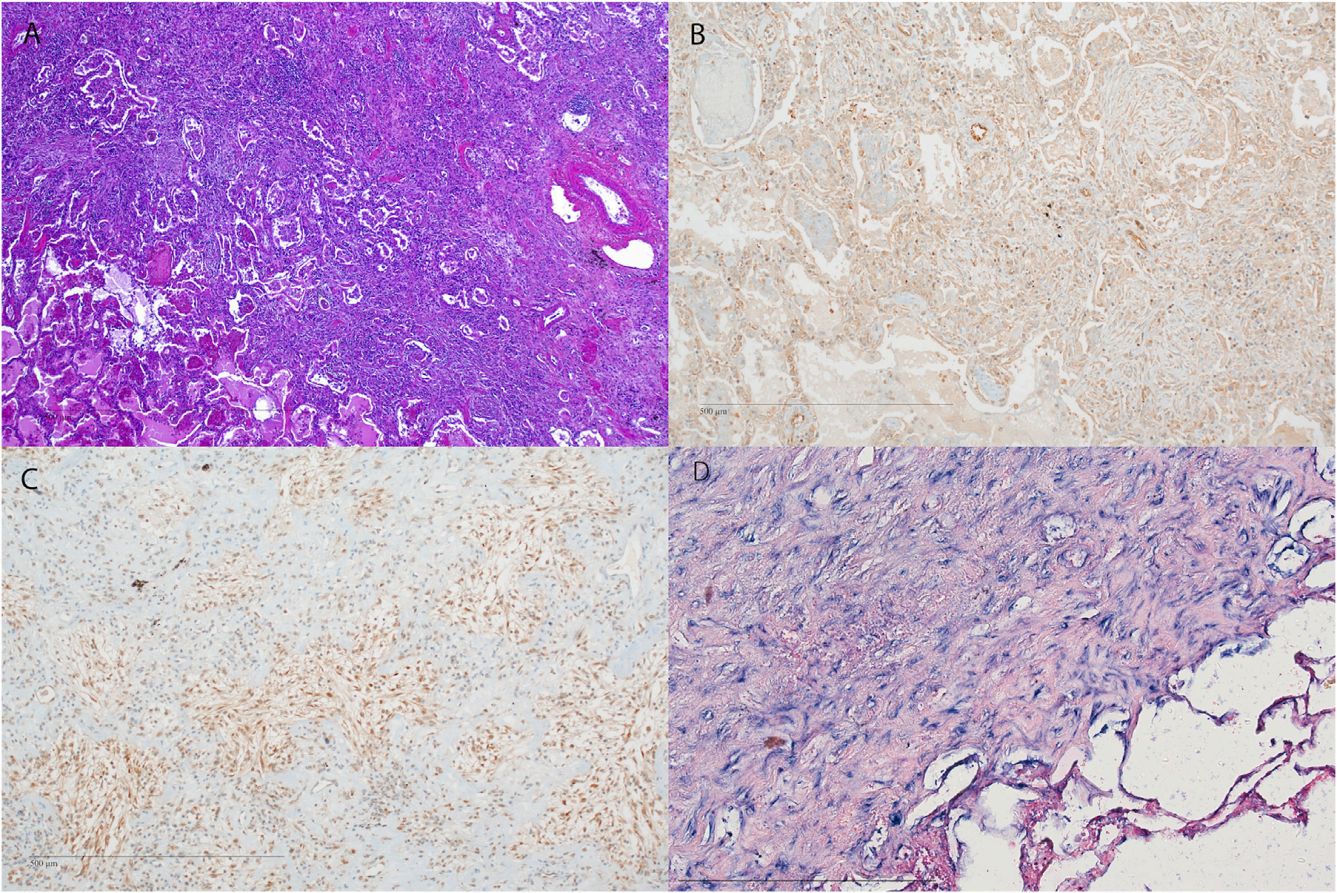
Representative images of fibrosis in RAS. **(A–D)** H&E staining of the pleura and subpleural parenchyma with fibrosis (original magnification ×40). **(B–D)** Immunohistochemistry and *in situ* hybridization of the corresponding HE **(A)** showing **(B)** NOTCH1, **(C)** pSMAD2,3 expression. **(A–D)** original magnification ×10). H&E = Hematoxylin and Eosin; miR-21 = microRNA-21, ISH = *in situ* hybridization.

Both, BOS (17, 100%) and RAS (6, 86%) specimens, showed strong expression of vimentin in peribronchiolar myofibroblasts, suggesting mesenchymal differentiation (Table 2; Figures 3, 4, Supplementary Figure S2). Notch-intracellular domain was positive in 12 (71%) BOS cases, predominantly in the cytoplasm of peribronchiolar myofibroblasts and bronchiolar epithelium, and in 4 (57%) RAS cases in the cytoplasm of interstitial fibroblasts. Finally, staining of p-SMAD 2/3 was positive in 7 (42%) BOS and 4 (57%) RAS specimens. pSMAD-2/3 expression was prevalent in interstitial fibroblasts as well as in the peribronchiolar myofibroblasts and bronchiolar epithelium in BOS allografts while in RAS allografts it was mainly present in interstitial fibroblasts (Table 2). β-catenin and E-cadherin stainings were negative in specimens of both phenotypes (Supplementary Figure S3). All four markers were negative in control tissue from donor lungs (Supplementary Figure S4). Correlations between IHC stainings of EMT markers and miR-21 ISH were calculated with Pearson correlation coefficients (Table 3). In BOS specimens, a strong positive correlation was found between miR-21 and p-SMAD 2/3 expression in the interstitium (*r* = 0.649, *p* = 0.006) as well as between miR-21 and NICD in the bronchiolar epithelium and myofibroblasts in the BO lesions (*r* = 0.827, *p* < 0.001). In RAS specimens, a positive correlation was found between miR-21 staining and NICD expression both in the fibroblasts in the interstitium (*r* = 0.837, *p* = 0.019) and peribronchial (*r* = 0.842, *p* = 0.018).

**TABLE 3:** Correlations.

Variables		MiR-21			
		BOS^a^ (*n* = 17)		RAS^b^ (*n* = 7)	
		*r*	*p*-value	*r*	*p*-value
Interstitium	Vimentin	0.564	0.023	0.000	1.000
	NICD^c^	0.417	0.096	0.837	0.019
	p-SMAD^d^ 2/3	0.649	0.006	0.230	0.620
Intra/Peribronchiolar	Vimentin	0.224	0.404	0.270	0.558
	NICD	0.827	0.0001	0.842	0.018
	p-SMAD 2/3	0.374	0.154	−0.258	0.576

a Bronchiolitis obliterans syndrome.

b Restrictive allograft syndrome.

c Notch intracellular domain.

d Sma and Mad-reated protein.

### Circulating miRNA-21 Before and After CLAD Onset

**FIGURE 5:**
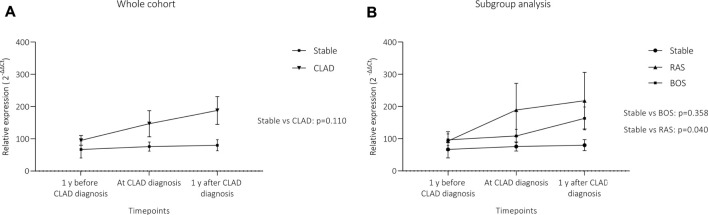
Differences in circulating miR-21 expression over time. **(A)** depicts the comparison between the whole CLAD cohort and stable patients, **(B)** shows results for CLAD subgroups. Although there was a visible trend towards higher miR-21 levels in the whole CLAD cohort, this did not reach the level of significance. However, when the two phenotypes were analyzed separately, RAS patients showed a significant increase in circulating miR-21 over time. MiR-21 expression is presented as 2^−∆∆Ct^ values (Mean + Standard error). Statistical significance was tested using a 2-way repeated measure ANOVA. **(A)** CLAD (*n* = 25) vs stable (*n* = 26); **(B)** BOS (*n* = 13), RAS (*n* = 12) and stable (*n* = 26).

Figure 5 summarizes analysis of miR-21 expression difference over time between the groups. CLAD patients showed a non-significant trend towards higher miR-21 expression over time compared to stable patients (Panel A, *p* = 0.110). Panel B shows results of subgroup analysis with RAS patients having a significant increase in serum concentration of miR-21 overtime as compared to stable patients (*p* = 0.040). No difference was observed between BOS patients and control patients (*p* = 0.358).

## Discussion

Lung transplantation is a well-established treatment for end-stage lung disease; however, long-term success is still impaired by chronic lung allograft dysfunction with a cumulative incidence of about 50% at 5 years after transplantation (1). CLAD pathophysiology is characterized by several stimuli which trigger graft remodeling and irreversible allograft fibrosis (11). Current evidence suggests the activation of TGF-β dependent and independent mechanisms as well as the role micro-RNAs acting as central regulators of EMT in CLAD (3, 6). Our study aimed to investigate the expression of miR-21 in CLAD and to correlate it with a set of key transcription factors of fibroproliferative processes. MiR-21 was expressed in most of the explanted CLAD allografts. Histologic miR-21 data were validated by serum analyses showing that RAS patients tend to have higher levels of circulating miR-21.

Micro-RNAs belong to the group of small non-coding-RNAs. They control post-translational gene expression by binding to mRNA and inducing its degradation or inhibiting its translation. They play a fundamental role in key biological processes including cell development, regulation of immunity and apoptosis (12). The pathogenic role of miR-21 in fibrotic processes has recently been highlighted. MiR-21 was found to be upregulated in lung tissue as well as serum of patients with idiopathic pulmonary fibrosis (13). In addition, miR-21 has been found to play a central role in the development of cardiac and renal fibrosis (14, 15). The transcription of miR-21 is under the control of several transcription factors (e.g., AP-1, SRF, p53, STAT3) (16), targets of miR-21 are manifold. It suppresses cell growth and invasiveness, induces cell cycle stop, inhibits matrix metalloproteases and other proteases, inhibits angiogenesis, cellular branching and migration (16). Moreover, it seems to be involved in an amplifying circuit to enhance TGF-β1 signaling and thus promote the progression of fibrotic lung diseases (6). Only recently, miRNAs were studied in lung transplantation. Xu et al. found a dysregulated set of miRNAs and their target genes in lung recipients with BOS and recipients who developed donor-specific antibodies (17). In mouse models of bronchiolitis obliterans, miR-21, miR-146, miR-20, miR-302, miR-19, miR-98, let-7a, miR-15a were altered in affected animals (18, 19). In 20 BOS patients, analysis of miR-144 showed a 4-fold increase in BOS patients with a parallel reduction of its target, TGF-β–induced factor homeobox 1 (TGIF1) (20). In our study, miR-21 expression was evaluated in 17 BOS and seven explanted RAS allografts. It was found upregulated both in fibroblasts of BO lesions as well as in the interstitial myofibroblasts of RAS specimens. Moreover, macrophages showed strong miR-21 staining. Levels of circulating miR-21 were then longitudinally investigated in lung recipients. Although not significantly significant, miR-21 levels tend to be higher in patients with an established CLAD diagnosis. Taken together, miR-21 may play a role in the fibrotic derangements of CLAD allografts and it might be used as a non-invasive diagnostic marker or serve as a therapeutic target.

Parenchymal injury and inflammation activate stromal fibroblasts, recruit circulating mesenchymal progenitor cells and induce EMT. Inflammatory milieu is the main contributor to excessive tissue remodeling and fibrosis. Macrophages are a potent source of TGF-β, which is one of the main contributors of EMT and fibroblast activation. EMT is the transdifferentiation process of epithelial cells into motile mesenchymal cells. It plays a role in embryonic development and wound healing but also contributes to pathological processes such as fibrosis and cancer progression. EMT is a complex phenomenon, which includes a crosstalk between signaling pathways and transcriptional, translational and post-translational regulation (21). Downregulation of E-cadherin destabilizes adherens junctions and promotes loss of the epithelial barrier function (21). The intermediate filament composition changes with the repression of cytokeratin and the activation of vimentin expression. This could also be confirmed in our study. Immunohistochemical stainings showed a complete absence of E-cadherin and a diffuse expression of vimentin in all explanted CLAD allografts. EMT is regulated by several signaling pathway. The most studied pathway is the TGF-β-SMAD pathway (22). TGF-ß1 up-regulation and SMAD3 activation have been previously described in BO lesion (18, 23, 24). Moreover, miR-21 plays an important role in SMAD-dependent TGF-β1 signal amplification (25). These findings could be confirmed in our patient cohort. High expression of phosphorylated form of SMAD 2/3 was found in the majority of CLAD samples. Concomitant miR-21 expression was confirmed by ISH. MiR-21 was also strongly stained in alveolar macrophages, known source of TGF-β. Alveolar macrophages were found to produce high levels of miR-21 containing liposomes, which induced EMT in tracheal epithelial cells through TGF-β1/Smad signaling pathway (26). Therefore, it is reasonable to hypothesize that alveolar macrophages play a central role in promoting fibroproliferative mechanisms by secreting exosomes containing fibrosis inducers such as cytokines and miRNAs. Though less studied, Wnt/β-catenin and Jagged-Notch signaling pathways also seem to be important in EMT induction (27-29). In our analysis, the active form of Notch was expressed in 69% of BOS and 42% of RAS allograft. This data suggests an active role of the Notch pathway in CLAD pathogenesis and further support the hypothesis that EMT is relevant in CLAD. On the contrary, expression of β-catenin was absent in both, BOS and RAS specimens.

We acknowledge there are several limitations of our study. Despite a relatively high number of re-transplantations performed in our institution, the sample size of lung specimens of RAS patients was small. Second, miR-21 was not measured in lung parenchyma of early-stages CLAD. Transbronchial biopsies have a low sensitivity for bronchiolitis obliterans or interstitial fibrosis (30), thus, they are not routinely performed in these patients. A panel investigation of other profibrinogenic and antifibrogenic microRNAs could have improved our mechanistic understanding of CLAD. Finally, miR-21 positive cells were only defined by their histomorphological appearance. Immunohistological co-stainings would have been required to confirm miR-21 expressing cells types.

In conclusion, this study could show that mir-21 is expressed both in tissue and serum in a large cohort of CLAD patients and its expression significantly increased in RAS patients over time. Moreover, its expression correlates with key markers of EMT. Further research is necessary to elucidate role miR-21 as a therapeutic target of CLAD.

## Data Availability

The datasets presented in this study can be found in online repositories. The names of the repository/repositories and accession number(s) can be found in the article/Supplementary Material.

## Capsule Summary Sentence

Long-term outcomes after lung transplantation are still hampered by the development of chronic lung allograft dysfunction (CLAD). Despite all efforts, the pathogenetic mechanisms of CLAD are not fully understood, however, fibroproliferation and epithelialto-mesenchymal transition have recently been described as important factors. Micro-RNAs are post-translational regulators of a variety of pathologic processes and miR-21 has been previously linked to fibrosis. To the best of our knowledge, our manuscript describes for the first time miR-21 expression in CLAD. We analyzed tissue samples of BOS and RAS allografts, as well as serum samples and could show that this miRNA is highly expressed in both CLAD subtypes. Of note, RAS patients had a significant increase in serum concentration of miR-21 overtime as compared to stable patients. Based on our results, miR-21 could serve as a future therapeutic target for CLAD.

## Supplementary Material

Supplementary Figure S1 Representative images of miR-21 ISH staining of alveolar macrophages (arrow) in BOS patients.

Supplementary Figure S2 Representative images of Vimentin staining in **(A)** BOS patients and **(B)** RAS patients [**(A,B)** original magnification 10×].

Supplementary Figure S3 Representative images of β-catenin and E-cadherin staining. in **(A–C)** β-catenin and E-cadherin in BOS patients and **(B–D)** β-catenin and E-cadherin in RAS patients [**(A,B)** original magnification 10×].

Supplementary Figure S4 Representative images of positive controls. **(A)** pSMAD2,3 **(B)** NOTCH1 **(C)** β-catenin **(D)** E-cadherin **(E)** Vimentin [**(A,B)** original magnification 10×].

## Abbreviations

CLAD, chronic lung allograft dysfunction; LTx, lung transplantation; BOS, bronchiolitis obliterans syndrome; RAS, restrictive allograft syndrome; EMT, Epithelial mesenchymal transition; TGF-β1, transforming growth factor-β1; miR-21, micro RNA-21; FFPE, formalin-fixed paraffin-embedded; COPD, chronic obstructive pulmonary disease; AMR, Antibody-mediated rejection; ACR, acute cellular rejection; LB, lymphocytic bronchiolitis; ISHLT, International society of heart and lung transplantation; ISH, *in situ* hybridization; IHC, immunohistochemistry; NICD, notch intracellular domain; SMAD, Sma and Mad-related protein.
