# Supplementary figures and images for: MiR-21 in Lung Transplant Recipients With Chronic Lung Allograft Dysfunction
Source: Transpl Int. 2022 Jan 13;35:10184. doi: 10.3389/ti.2021.10184 (PMC8842266; doi:10.3389/ti.2021.10184)

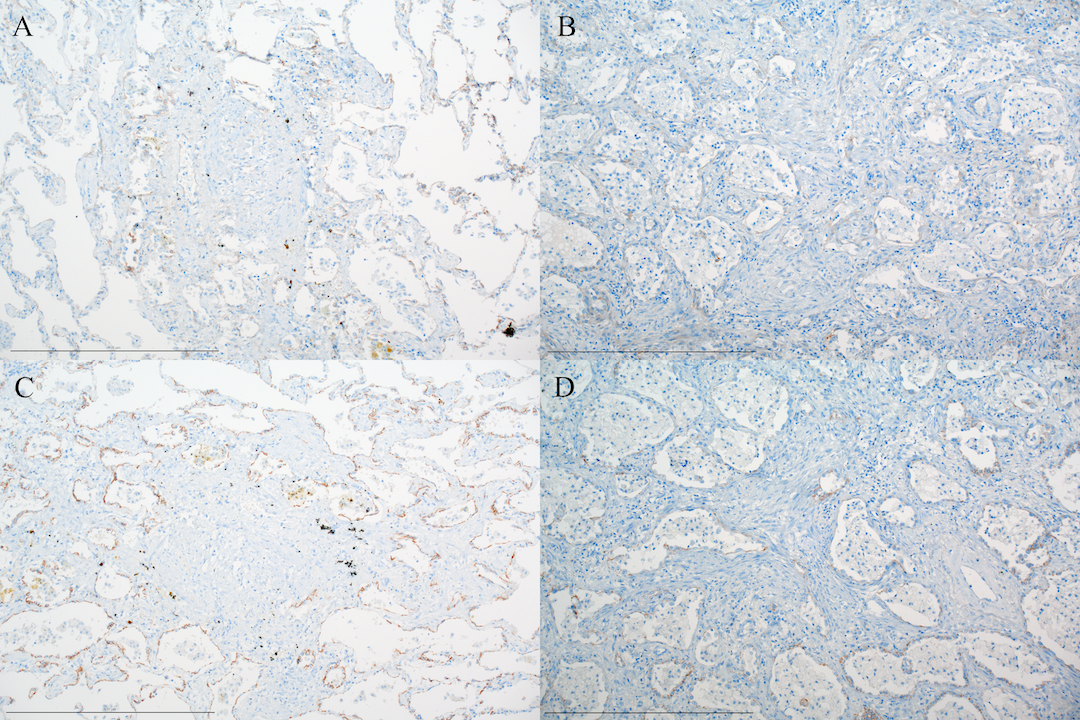

Supplement: Supplementary file 1 [file Image3.TIF]

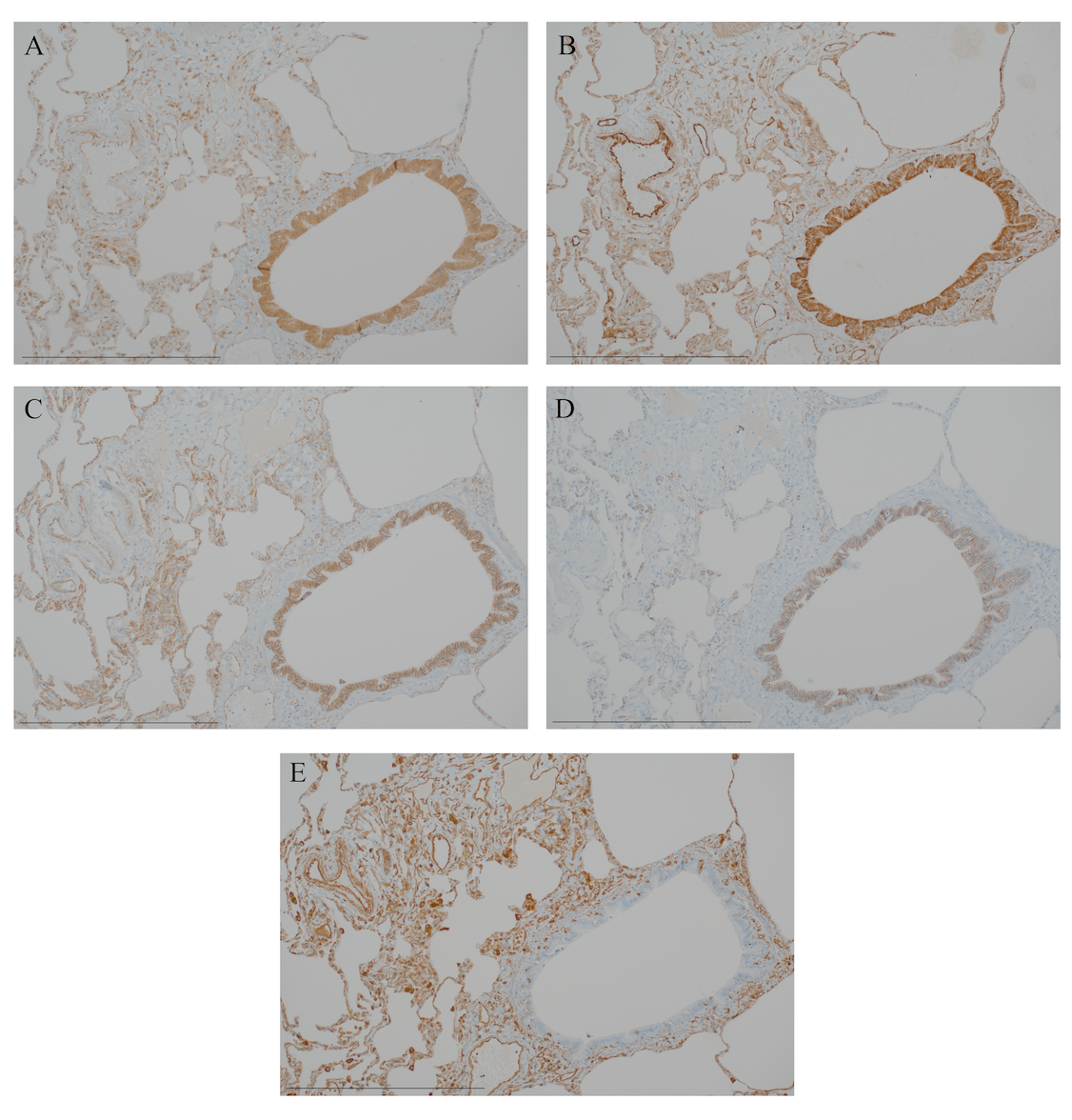

Supplement: Supplementary file 2 [file Image4.TIF]

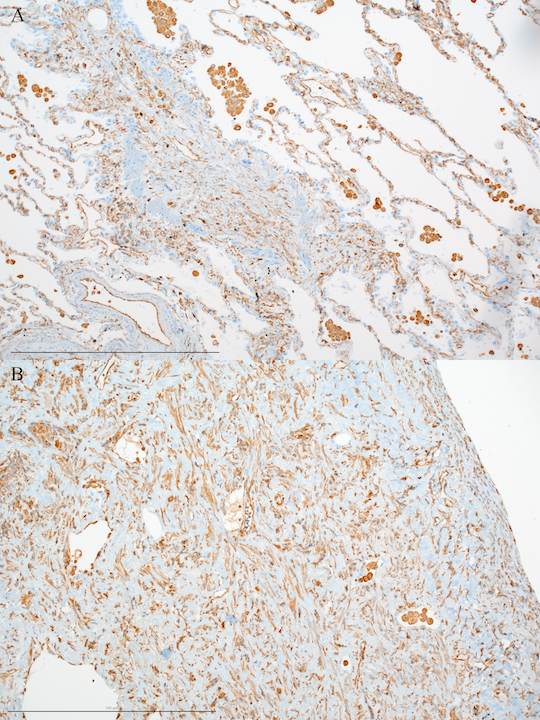

Supplement: Supplementary file 3 [file Image2.TIF]

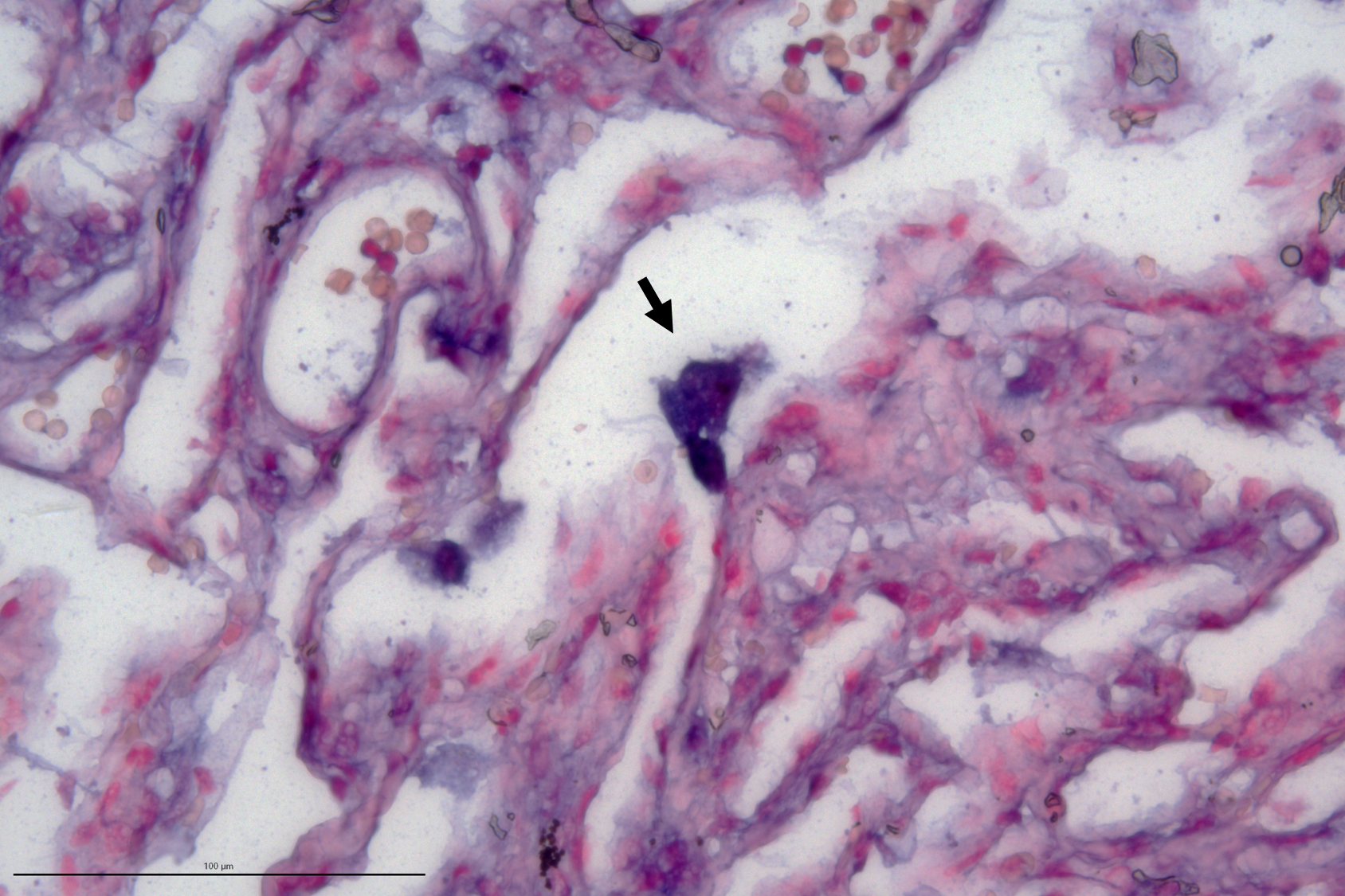

Supplement: Supplementary file 4 [file Image1.TIF]
